# Government Health Expenditure, Economic Growth, and Regional Development Differences—Analysis Based on a Non-parametric Additive Model

**DOI:** 10.3389/fpubh.2022.925910

**Published:** 2022-07-11

**Authors:** Yuxiao Wang, Chunhai Tao, Qizhe Xiong

**Affiliations:** School of Statistics, Jiangxi University of Finance and Economics, Nanchang, China

**Keywords:** government health expenditure, economic growth, regional development differences, non-parametric additive model, non-linear effects

## Abstract

Government health expenditure plays an important role in guaranteeing people's livelihood and in improving the quality of human capital, and it also plays an important role in affecting economic development. In order to characterize the specific trend of government health spending affecting economic growth, and analyze the impact difference in different regions, this paper uses a non-parametric additive model to analyze the impact of government health expenditure on economic development and regional development differences based on three aspects: linear, non-linear and comprehensive effects. From the perspective of linear effects, the results show that the proportion of government health expenditure to GDP nationwide and in the western regions has a positive impact on economic development, while the impact in the eastern and central regions is negative. From the perspective of non-linear effects, in the national and in the eastern, central and western regions, the proportion of government health expenditure to GDP has a significant non-linear impact on economic development. From the perspective of comprehensive effects, the proportion of government health expenditure to GDP has a significant non-linear positive effect on economic development nationwide and in all regions. In addition, the proportion of fixed assets investment to GDP, the proportion of exports to GDP, and the proportion of residents' income to GDP promote non-linear effects to different extents of non-linear promotion, but there are slight differences in different regions. Based on estimation results, the paper recommends that the government further expands the proportion of government health expenditure in GDP, promotes investment in fixed assets, stimulates exports in the eastern region, and continues to implement the western support policy.

## Introduction

Report on the work of the government (1) pointed out that “Setbacks in economic globalization, challenges to multilateralism, shocks in the international financial market, and especially the China-US economic and trade frictions, had an adverse effect on the production and business operations of some companies and on market expectations. What we faced were severe challenges caused by the growing pains of economic transformation. An interlacing of old and new issues and a combination of cyclical and structural problems brought changes in what was a generally stable economic performance, some of which caused concern. What we faced was a complicated terrain of increasing dilemmas. We had multiple targets to attain, like ensuring stable growth and preventing risks, multiple tasks to complete, like promoting economic and social development, and multiple relationships to handle, like that between short-term and long-term interests. And the difficulty of making policy choices and moving work forward increased markedly.” China's economic development faces a complex and severe external environment, as well as economic challenges due to intertwining contradictions and structural transformation, bringing great difficulties for the steady advancement of China's economy. Therefore, in order to achieve sustained and steady economic growth, we need to begin with all aspects of promoting economic development. Over the years, economic development has always been an important factor influencing national development and social stability. It is the focus of the country and its people, and it is also the focus of many scholars. Regarding existing research at home and abroad, some studies focus on human capital and economic development, such as Xu and Li (2), who studied the relationship between innovative human capital and provincial economies. Zeqiraj et al. (3) found that human capital has a great impact on economic growth. Wang (4) found that human capital in Northeast China plays a decisive role in economic growth. Chen et al. (5) found that human capital has a decreasing impact on the economy in China from east to the west in China. Liu and Xia (6) found that an improvement in the human capital level has a significant role in promoting the quality of economic growth in a province, but has a negative impact on the quality of economic growth in neighboring provinces.

Some studies focus on technological development and economic development. For example, Mensah et al. (7) studied the impact of technological innovation on 28 economies of the Organization for Economic Co-operation and Development (OECD) from 2000 to 2014. Singh et al. (8) studied the relationship between grassroots technological innovation and sustainable development. Ma et al. (9) studied the relationship between urban infrastructure, technological innovation and regional economic development. Wang (10) studied technological innovation in a regional circular economy. Li (11) carried out research on the impact of agricultural technological innovation on agricultural economic development. Song (12) analyzed the relationship between technological innovation and economic growth in Shandong Province.

Although there are many studies on economic development, there are few studies on economic development and government health expenditure, most of which focus on the relationship between medical insurance, personal medical expenditure, and government fiscal expenditure, such as Benoît and Coron (13), who analyzed the financialization of private health insurance in France. Karunaratna et al. (14) studied the effectiveness of compulsory social insurance schemes in regard to the financial burden of patients. Yao et al. (15) analyzed the impact of microhealth insurance on maternity-related costs. Only a few studies have investigated the relationship between health expenditure and economic growth, such as Kumar et al. (16), Zhao et al. (17), Atems (18), Lee et al. (19), Halici-Tülüce et al. (20), Wang and Lee (21), and Wang (22). Regarding the relationship between health expenditure and growth, most studies only analyze only its linear effects, while the increase of government health expenditure can promote the increase of medical and health resources, thereby creating more and better medical conditions for the residents, which can promote the improvement of the health level of human resources and prolong the life expectancy. It can further increase average working hours and socioeconomic benefits. So it may have a non-linear impact. Only a few analyze the non-linear effects, the specific form of influence is not analyzed in detail; thus, its specific trend is still not accurately captured. The study aims to fill in the gaps in extant scholarship.

In 1985, the additive model proposed by Stone (23) does not need to specifically set the relationship between variables. It does not limit the relationship between variables to a specific form, but can automatically fit according to the observed values. It makes the relationship between variables more accurate and specific. The non-parametric additive model incorporates a linear part on the basis of the additive model. It not only can describe the linear relationship between variables, but also can specifically describe the specific non-linear effects between variables. Although the fitting curve of the non-parametric additive model cannot be represented by a function, and the parameter estimates cannot be given, this prediction effect is better than that of the pure parametric model. Therefore, the paper adopts a non-parametric additive model to analyze the impact of government health expenditure on economic development.

As an important expenditure of government finance, government health expenditure is an important factor affecting the development of the health industry of a nation and the health of its people. It also has a non-negligible impact on economic development. How and how much does government health expenditure impact economic development? This study is guided by this important research question. This study conducts a detailed analysis of this issue to meet the following objectives:

The paper not only analyzes the linear relationship between government health expenditure and economic development but also examines the trend of its non-linear influence, clarifying the impact of government health expenditure on economic development.

It uses a non-parametric additive model to further explore the impact of government health expenditure on economic growth, analyzes the differences in the impact of different regions, and supplements and expands the research methods related to this problem.

It combine linear effect analysis, non-linear effect analysis and comprehensive effect analysis with novel angles, supplements and improves the theoretical research related to this problem, and provided theoretical support for macroeconomic regulation and long-term, stable economic development.

Following this introduction, this paper has five parts: the first part presents the theory and the model. The second part describes the selection, use and processing of the indicators and data. The third part tests the applicability of the model. The fourth part conducts a discussion and analysis of the estimation results. The fifth part summarizes the conclusions of the paper and proposes targeted policies.

## Theory and Model

### Theoretical Analysis

The government can have different impacts on society through health investment, which then directly or indirectly connects with economic development. In the current social development environment, Tao and Wang (24) analyzed the effect of government health expenditure on economic development based on the following aspects:

The government promotes the development and improvement of the medical and health market by increasing financial and medical investment and medical system reform, thereby creating a fair, orderly and efficient medical and health market, creating a sound medical and health economic system and improving medical institutions and medical care. The quality of personnel services promotes the effective improvement of medical technology and work efficiency, greatly improves the medical quality of hospitals, promotes the economic income of hospitals and medical personnel, and promotes economic efficiency.

Increases in financial and medical investment promote increases in medical and health resources, promote the scale of medical resources, and thus create more and better medical conditions for residents. Such increases can promote the improvement of human resource health and extend life expectancy, and workers can serve society. The increase in the average labor time provided will increase social and economic benefits, and it can promote the improvement of human resources technology and creativity, promote the innovative development of enterprises and industries, and effectively improve the rapid development of the economy.

Financial labor input can promote the employment of the labor force, and it can provide workers with stable and sustained economic income. Furthermore, ensuring the quality of life of workers can appropriately increase the disposable income of workers and increase the purchasing power of residents, thereby increasing consumer demand, promoting the development of consumer industries, and stimulating domestic demand, thus effectively promoting economic development.

**Figure 1:**
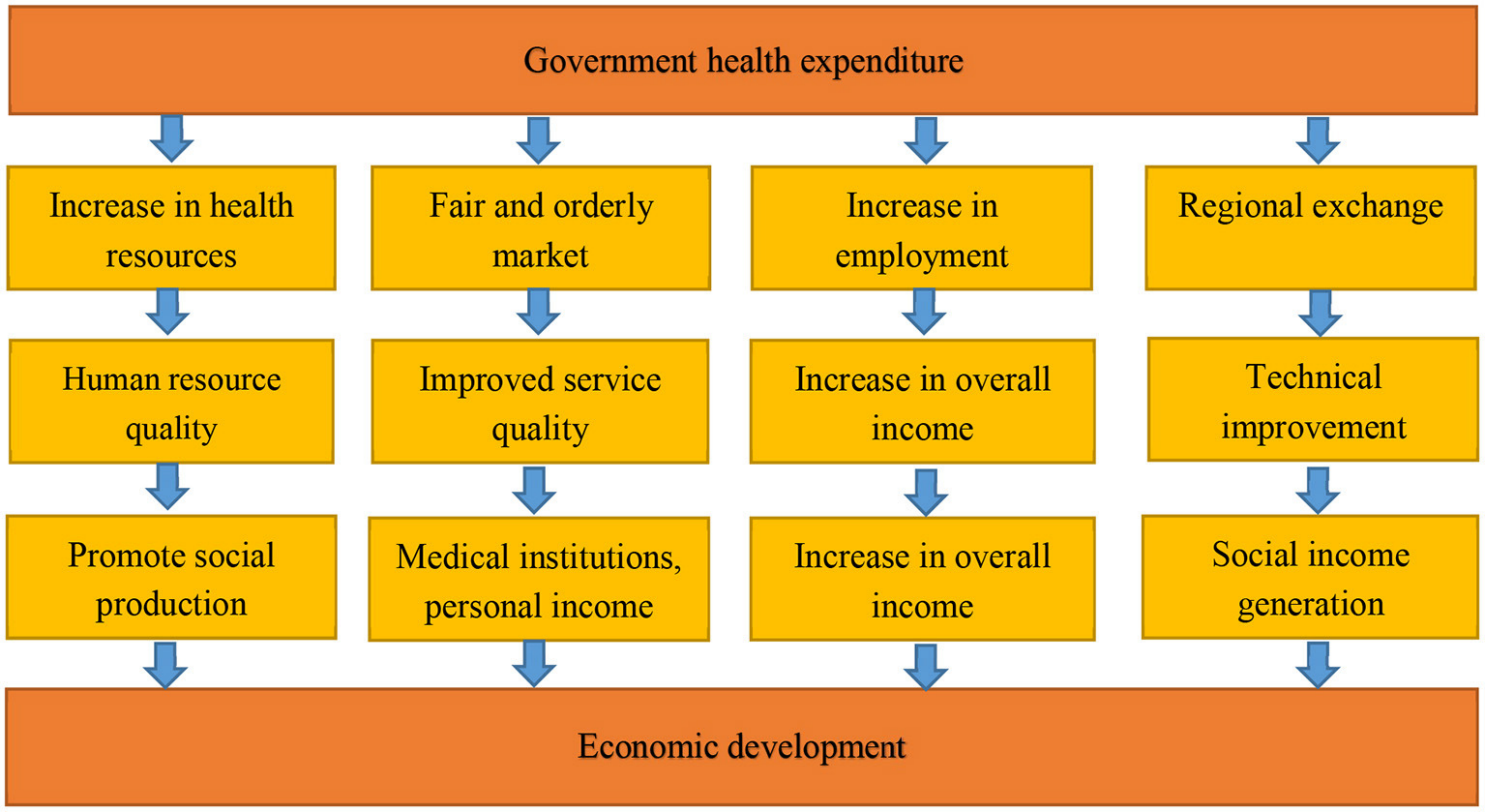
Mechanistic conduction route.

The government health expenditures in various regions affect each other and thus have an impact on regional economic development (as shown in Figure 1).

Government health expenditure directly and indirectly affects economic development through various social connections. Therefore, government health expenditure will not have a simple linear relationship with economic development, and because of the differences in government health expenditure in different regions, the impacts on different regions will be different.

### Model Introduction

Linear models are simple, intuitive, and easy to understand. However, in real life, the role of variables is usually not linear, and linear assumptions may not meet actual needs and may even be contrary to actual conditions. The non-parametric additive model is an extension of the additive model proposed by Stone (23); it is a freely flexible statistical model that detects non-linear effects between variables. The classical linear regression model assumes that the dependent variable *y* and the independent variables *x*_1_, *x*_2_, ⋯*x*_*n*_ are linear:


(1) $$\begin{array}{l} y\;=\;\beta_{0}\;+\;\beta_{1}x_{1}\;+\;\beta_{2}x_{2}\;+\;\cdots \;+\;\beta_{n}x_{n} \end{array}$$

where β_0_ is the intercept term, and β_1_, β_2_, ⋯ , β_*n*_ are the estimated parameters for each variable.

The generalized additive model is an extension of the linear model:


(2) $$\begin{array}{l} y\;=\;s_{0}\;+\;s_{1}\left(x_{1}\right)\;+\;s_{2}\left(x_{2}\right)\;+\;\cdots \;+\;s_{n}\left(x_{n}\right) \end{array}$$

where *s*_0_ is the intercept term, and *s*(·) is the non-parametric function.

It is important to effectively understand the intrinsic relationship between the explanatory variables and the explained variables and to enhance the interpretability and visualization of the model. So we incorporate the linear part on the basis of the additive model to form a new non-parametric additive model:


(3) $$\begin{array}{l} y\;=\;\alpha \;+\;\overset{n}{\underset{i=1}{\sum }}\beta_{i}x_{i}\;+\;\overset{n}{\underset{i=1}{\sum }}s\left(x_{i}\right) \end{array}$$

where $\alpha +\overset{n}{\underset{i=1}{\sum }}\beta_{i}x_{i}$ is the linear part of the model, and $\overset{n}{\underset{i=1}{\sum }}s\left(x_{i}\right)$ is the non-linear part of the model.

Based on the above model construction ideas, this article sets the following empirical model:


(4) $$\begin{array}{l} avegdp=\alpha +\beta_{1}RDhealth+\beta_{2}RDassets+\beta_{3}RDexport\;\\ \;+\beta_{4}RDincome+\beta_{5}lDurban+\beta_{6}lDold\;\\ \;+s(RDhealth)+s(RDassets)+s(RDexport)\;\\ \;+s(RDincome)+s(lDurban)+s(lDold)+\varepsilon \end{array}$$

## Variable Description and Data Processing

### Variable Selection

There are many studies at home and abroad on economic development, but this study mainly focuses on domestic economic development research; thus, it summarizes the status of domestic economic development and the conclusions of existing economic development research because this research focuses on the impact of government health expenditure on economic development. There are many factors influencing the economic growth, while the important factors are capital, labor, exports, income level, urbanization, etc. Fixed asset investment further affects economic growth through the competitive effect, the promotion of technological progress, the adjustment of industrial structure, and the externality, inducement, and orientation of public investment (25). After the population enters old age, its working time decreases, and the social and economic burden increases, which will have a certain inhibitory effect on economic growth (26–28). The increase in export volume will increase the demand for products and services, drive domestic production, increase employment, improve foreign trade exchanges, and increase market vitality, which is conducive to economic growth (29). Changes or differences in residents' income can be important to the economy (30, 31). Urbanization is conducive to promoting population agglomeration in cities, further generating economies of scale. And market demand will grow rapidly and diversify, which is conducive to promoting specialized division of labor and improving economic efficiency. The education level and health level of the people will be improved, and excellent human resources will be provided for economic development. In addition, over or under-concentration can be very costly in terms of productivity growth (32–34). We choose fixed assets investment to reflect capital, the proportion of the elderly population to reflect labor, the export volume to reflect the export situation, residents' income to reflect the income level, and urbanization to reflect the level of urbanization. Therefore, per capita GDP was selected as the explained variable, and the proportion of government health expenditure to GDP was selected as the main explanatory variable. The proportion of fixed assets investment to GDP, the proportion of exports to GDP, the proportion of residents' income to GDP, the level of urbanization, and the proportion of the elderly population were also estimated as covariates in the model. The specific variables are explained in Table 1.

**Table 1:** Explanation of the variables.

**Variable**	**Symbol description**	**Variable interpretation**
Per capita GDP	Avegdp	Gross domestic product/total population
The proportion of government health expenditure to GDP	RDhealth	Government health expenditure/gross domestic product
The proportion of fixed assets investment to GDP	RDassets	Total fixed assets investment/gross domestic product
The proportion of exports to GDP	RDexport	Total exports/gross domestic product
The proportion of residents' income to GDP	RDincome	Total resident income/gross domestic product
The level of urbanization	lDurban	Permanent resident population/total population
The proportion of the elderly population	lDold	Elderly population/total population

### Data Processing

The data for each variable selected by the study mainly come from the China Statistical Yearbook, China Population and Employment Statistics Yearbook, China Financial Yearbook, etc. The data from 31 provinces (municipalities, autonomous regions) during 1996–2017 are selected. For the study sample, the country is divided into three regions: the eastern, central and western regions. The eastern region includes 11 provinces (cities and autonomous regions): Beijing, Tianjin, Hebei, Liaoning, Shanghai, Jiangsu, Zhejiang, Fujian, Shandong, Guangdong, and Hainan. The central region includes 8 provinces (municipalities and autonomous regions): Shanxi, Jilin, Heilongjiang, Anhui, Jiangxi, Henan, Hubei, and Hunan. The western region includes 12 provinces (municipalities and autonomous regions): Inner Mongolia, Guangxi, Chongqing, Sichuan, Guizhou, Yunnan, Tibet, Shaanxi, Gansu, Qinghai, Ningxia, and Xinjiang. To eliminate the influence of the price level, the index data are reduced by the index of each variable in 1995. The BP test shows that there is heteroscedasticity. To avoid the influence of heteroscedasticity on the model results, the logarithm of the data for each variable is processed before model estimation.

## The Applicability Test

### Normality Test

**Figure 2:**
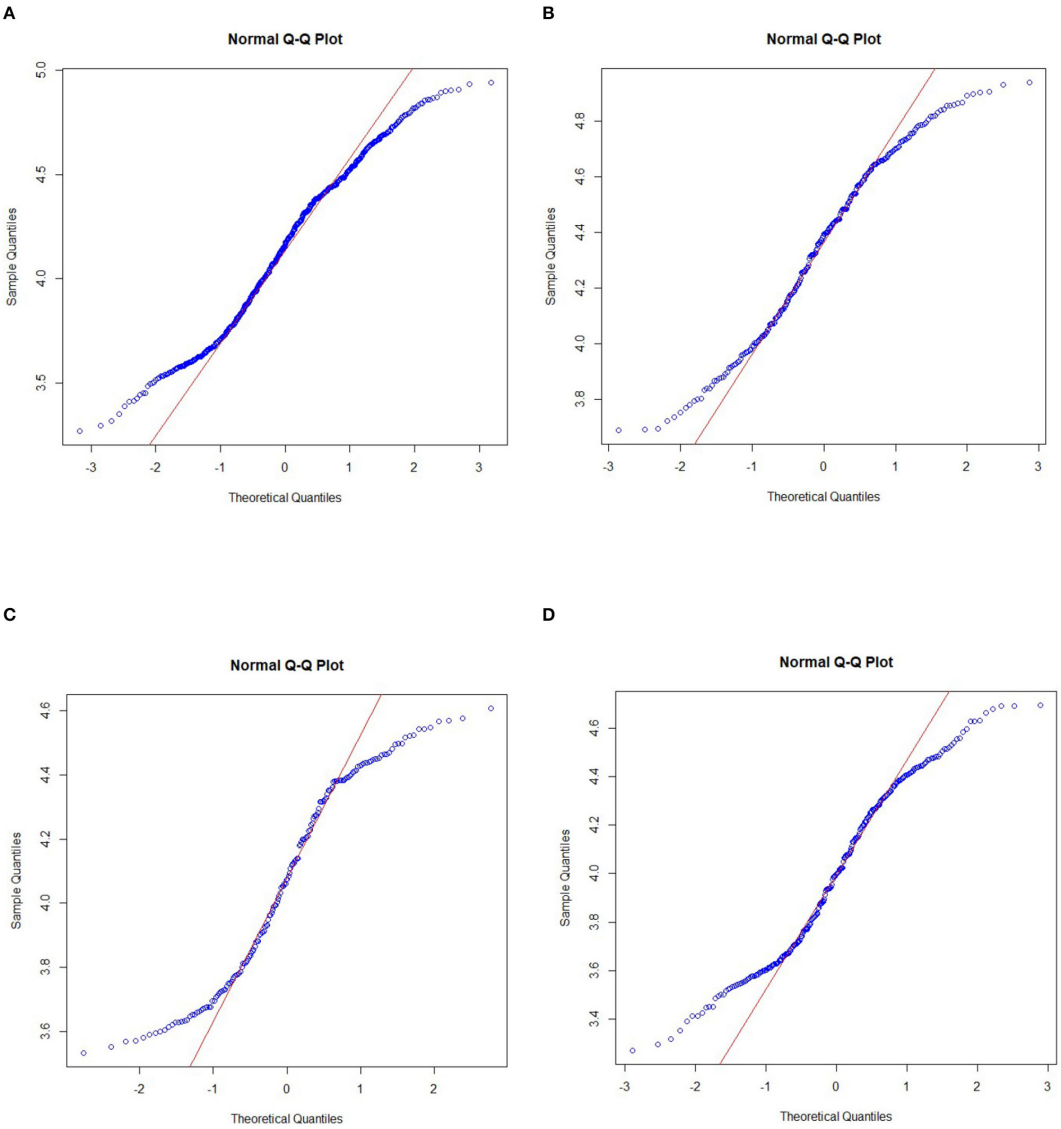
Q–Q plot of the normality test for the whole country and the eastern, central and western regions. **(A)** National Q–Q plot, **(B)** Eastern region Q–Q plot, **(C)** Central region Q–Q plot, and **(D)** Western region Q–Q plot.

To confirm whether the non-parametric additive model is suitable for analysis, the normality of the explained variable needs to be tested before using the model for regression estimation. Therefore, the normality of per capita GDP is first tested. The Q–Q plot and result statistics of the normality test are shown in Figure 2 and Table 2, respectively.

**Table 2:** Normality test statistical results for the nation and the eastern, central, and western regions.

	**Nation**	**Eastern region**	**Central region**	**Western region**
Skewness	−0.0566	−0.1618	−0.0527	0.0531
Kurtosis	2.0774	1.9793	1.6166	1.8362
W	0.9786	0.9692	0.9328	0.9604
*P*	1.99 × 10^−8^	4.265 × 10^−5^	2.621 × 10^−7^	1.223 × 10^−6^

From the results of the normality test Q–Q plot and the test statistics, per capita GDP does not conform to the normal distribution in the nation or the eastern, central and western regions, showing a distinct skewed distribution. Therefore, the distribution conforms to the use of the non-parametric additive model.

### Collinearity Test

Before using the model for analysis, in addition to the normality of the explained variables, the collinearity between the explanatory variables needs to be tested. If the model has obvious collinearity problems, the variance and standard error of the regression results will increase, the confidence interval will be expanded, and the model's accuracy will be reduced. Judging whether there is a collinearity problem between explanatory variables generally involves comparing the correlation coefficient R of an explanatory variable with other explanatory variables with a value of 0.5 (or comparing *R*^2^ with 0.25). When the absolute value of the correlation coefficient *R* > 0.5, there is a collinearity problem between the variables. When the absolute value of the correlation coefficient *R* <0.5, the collinearity problem between the explanatory variables is considered to be low or non-existent and may be ignored. The results of the collinearity test include three indicators: the maximum value (worst), the observed value (observed), and the estimated value (estimate). The estimated results of the three indicators for the collinearity test of each explanatory variable are shown in Table 3.

**Table 3:** Correlation coefficient test.

	**RDhealth**	**RDassets**	**RDexport**	**RDincome**	**lDurban**	**lDold**
**Worst**						
RDhealth	1	0.65	0.33	0.07	0.18	0.15
RDassets	0.65	1	0.37	0.17	0.25	0.17
RDexport	0.33	0.37	1	0.26	0.38	0.18
RDincome	0.07	0.17	0.26	1	0.27	0.18
lDurban	0.18	0.25	0.38	0.27	1	0.32
lDold	0.15	0.17	0.18	0.18	0.32	1
**Observed**						
RDhealth	1	0.58	0.15	0.04	0.13	0.10
RDassets	0.62	1	0.13	0.14	0.04	0.11
RDexport	0.17	0.24	1	0.18	0.33	0.14
RDincome	0.03	0.04	0.12	1	0.22	0.16
lDurban	0.05	0.15	0.31	0.15	1	0.24
lDold	0.04	0.03	0.12	0.13	0.29	1
**Estimate**						
RDhealth	1	0.54	0.17	0.04	0.13	0.09
RDassets	0.50	1	0.15	0.10	0.06	0.08
RDexport	0.18	0.14	1	0.14	0.24	0.12
RDincome	0.02	0.10	0.11	1	0.16	0.12
lDurban	0.05	0.19	0.26	0.12	1	0.23
lDold	0.04	0.11	0.10	0.10	0.22	1

It can be seen from the results of the three indicators that the correlation coefficient between the proportion of fixed assets and the proportion of government health expenditure exceeds 0.5; the other correlation coefficients are all below 0.5, basically passing the test. So non-parametric additive model estimation can be performed.

## The Analysis and Discussion of the Empirical Results

### National and Regional Model Estimation

This part uses the non-parametric additive model to analyze the linear, non-linear and comprehensive effects for the nation and the eastern, central and western regions.

#### Linear Results

Table 4 shows the results of the linear partial estimation for the nation and the eastern, central and western regions. It can be seen from the table that the proportion of government health expenditure for the whole country and for the western region has a positive impact on economic development. However, government health expenditure in the eastern and central regions has a negative impact on economic development. The results nationwide and for all regions are not significant. From the linear results, the extent of government health expenditure has different extents of impact on economic development in different regions.

**Table 4:** Estimation results of linear part.

	**Nation**		**Eastern region**		**Central region**		**Western region**	
	**Coefficients**	***P*-value**	**Coefficients**	***P*-value**	**Coefficients**	***P*-value**	**Coefficients**	***P*-value**
RDhealth	0.0177	0.218	−0.0156	0.6	−0.0041	0.62	0.0580	2 × 10^−16^
RDassets	0.6162	2 × 10^−16^	0.5682	2 × 10^−16^	0.6365	2 × 10^−16^	0.6592	2 × 10^−16^
RDexport	0.2921	2 × 10^−16^	0.4281	2 × 10^−16^	0.2706	2 × 10^−16^	0.2494	2 × 10^−16^
RDincome	0.6372	2 × 10^−16^	0.5787	2 × 10^−16^	0.6424	2 × 10^−16^	0.6657	2 × 10^−16^
lDurban	0.6537	2 × 10^−16^	0.6793	2 × 10^−16^	0.6171	2 × 10^−16^	0.5437	2 × 10^−16^
lDold	0.3391	2 × 10^−16^	0.3563	2 × 10^−16^	0.3588	2 × 10^−16^	0.3298	2 × 10^−16^
Intercept	0.3691	2 × 10^−16^	0.3654	2 × 10^−16^	0.3861	2 × 10^−16^	0.3744	2 × 10^−16^

In addition to the proportion of government health expenditure, the proportion of fixed assets investment to GDP, the proportion of exports to GDP, the proportion of residents' income to GDP, the level of urbanization, and the proportion of the elderly population on economic development have a positive impact on the nation and on the eastern, central and western regions. All these results passed the significance test. From the linear estimation results, in addition to the proportion of government health expenditure, other explanatory variables have a significant positive impact on the economic development of each region.

#### Non-linear Results

**Figure 3:**
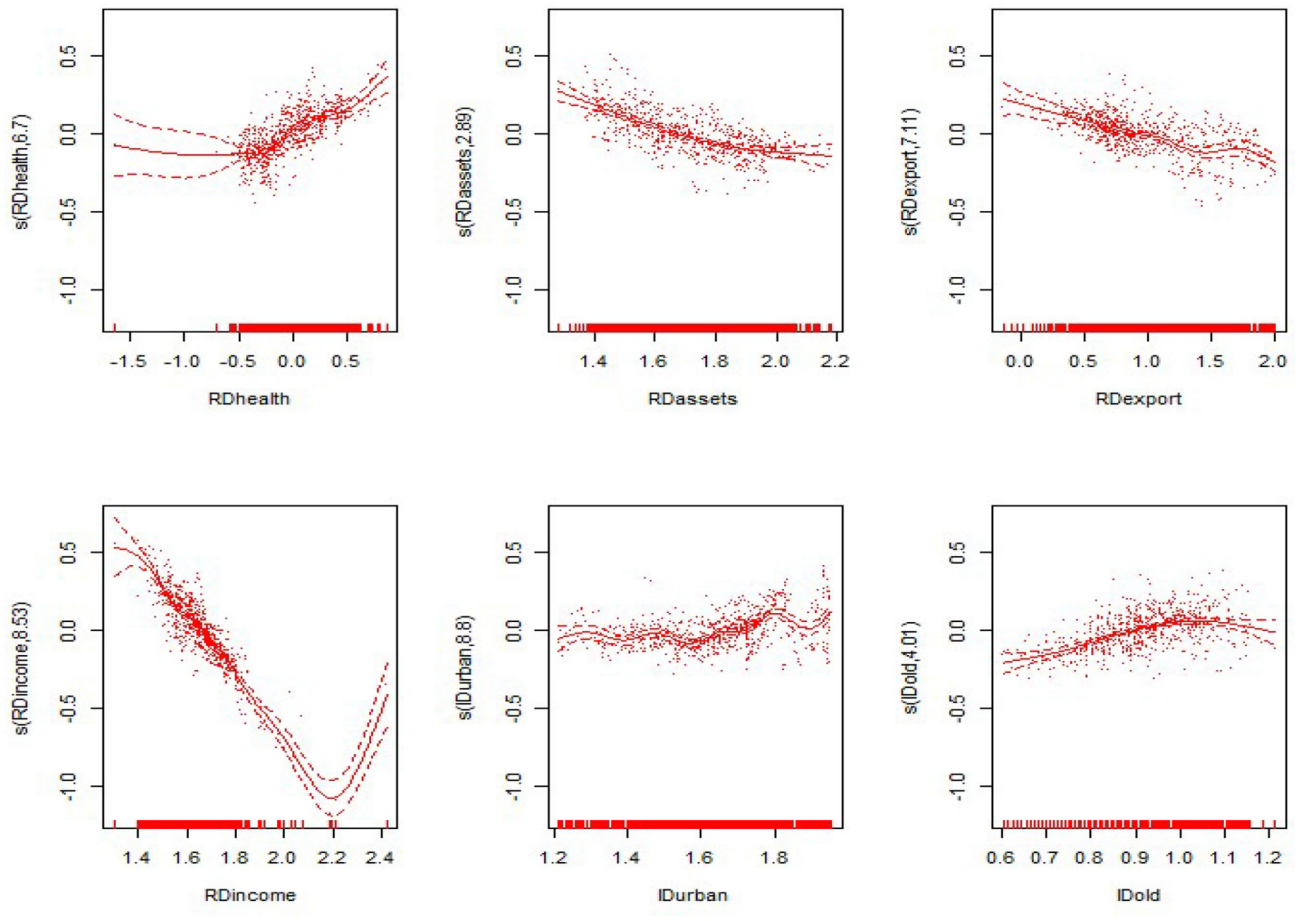
Non-linear effects of the explanatory variables on per capita GDP in the nation.

**Figure 4:**
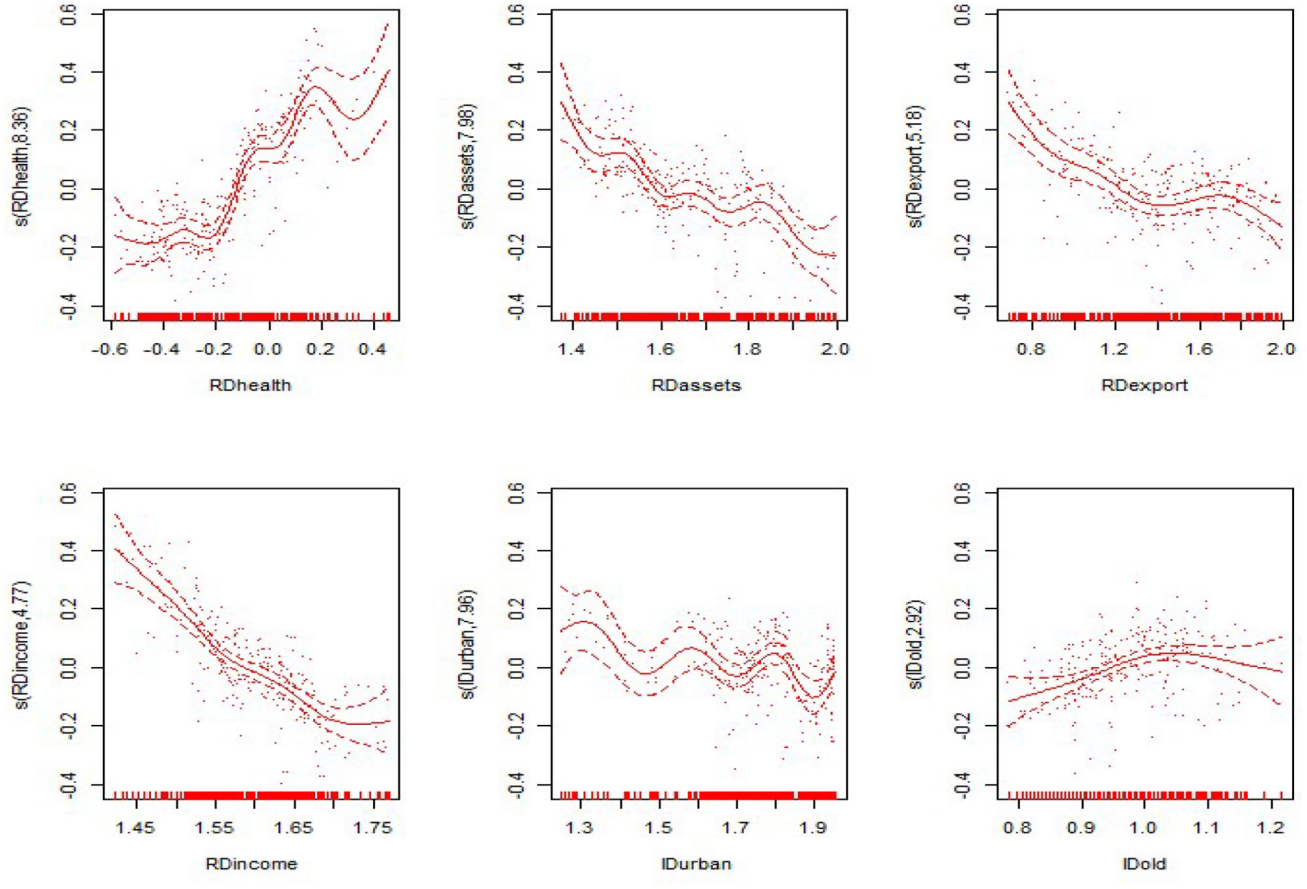
Non-linear effects of the explanatory variables on per capita GDP in the eastern region.

**Figure 5:**
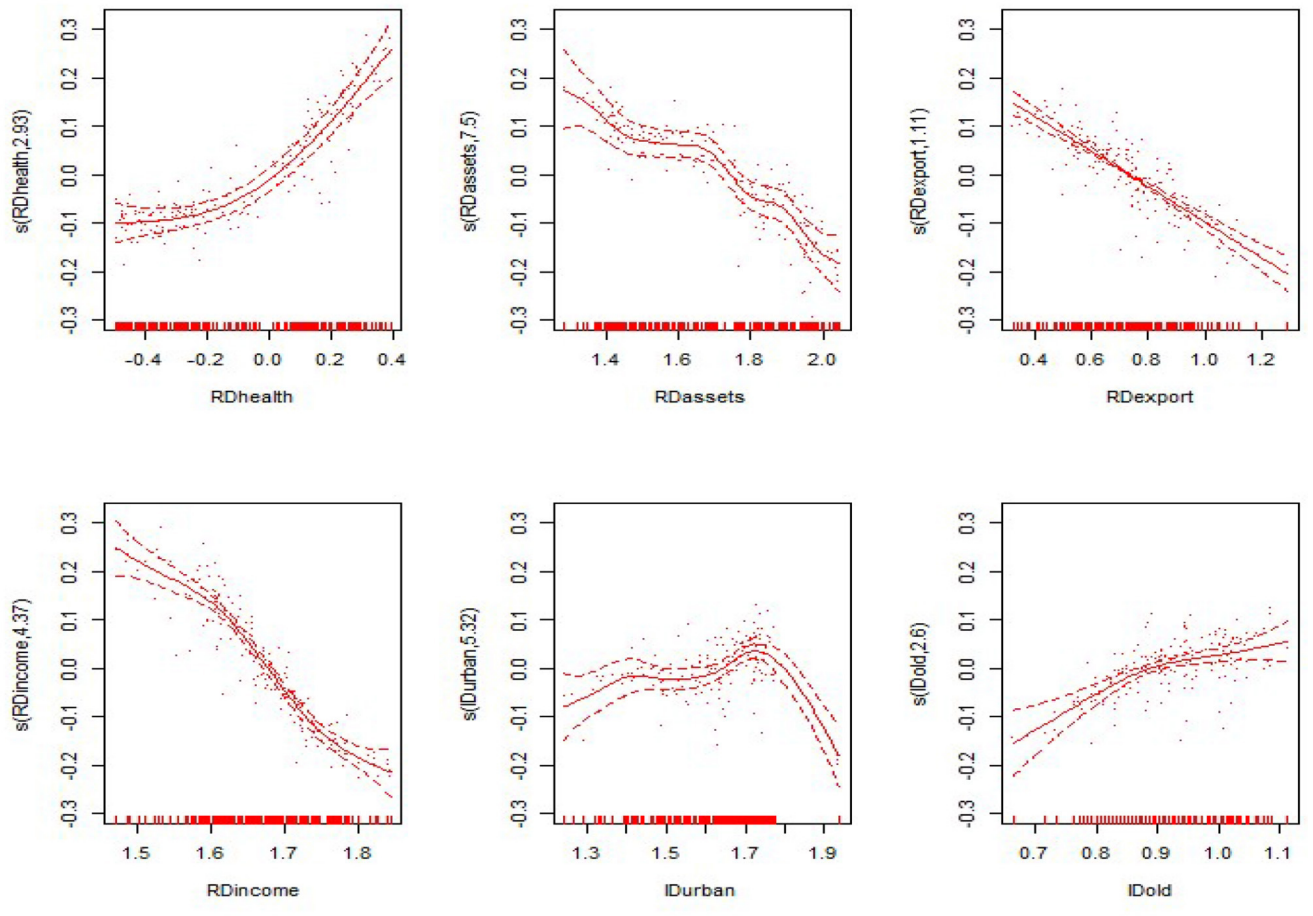
Non-linear effects of the explanatory variables on per capita GDP in the central region.

**Figure 6:**
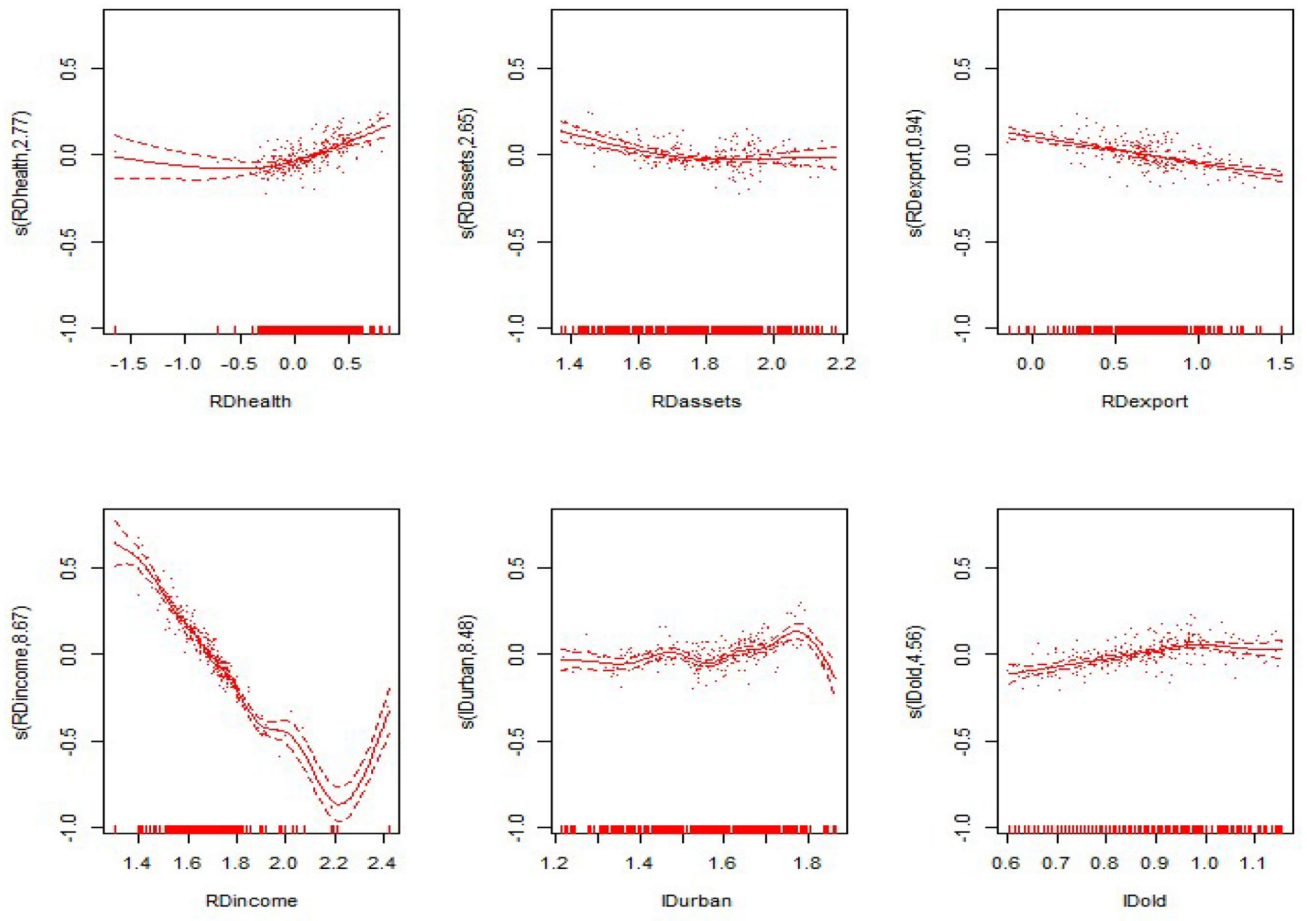
Non-linear effects of the explanatory variables on per capita GDP in the western region.

From the non-linear estimation results of the model, the model estimates the optimal smoothing degree of each explanatory variable and gives the *F*-value and the *P*-value of each variable (Table 5 evaluates only the degrees of freedom and the *P*-value). As seen from Table 5, the non-linear estimates of the proportion of government health expenditure to GDP, the proportion of fixed assets investment to GDP, the proportion of exports to GDP, the proportion of residents' income to GDP, the level of urbanization, and the proportion of the elderly population were all significant at the 0.1 level. This shows that all explanatory variables have significant non-linear effects on the nation and the eastern, central and western regions. The non-linear effects of various explanatory variables on the economic development of the nation and the eastern, central and western regions are shown in Figures 3–6. The solid line in the figures represents the non-linear effect of each explanatory variable on economic development, the dotted line represents the 95% confidence interval, and the scatter represents the observed value of the variable.

**Table 5:** Estimation results of non-linear part.

	**Nation**		**Eastern region**		**Central Region**		**Western region**	
	**Edf**	***P*-value**	**Edf**	***P*-value**	**Edf**	***P*-value**	**Edf**	***P*-value**
RDhealth	6.695	2 × 10^−16^	8.357	2 × 10^−16^	2.934	2 × 10^−16^	2.767	2 × 10^−8^
RDassets	2.889	2 × 10^−16^	7.977	1.04 × 10^−10^	7.499	1.02 × 10^−10^	2.650	8.61 × 10^−6^
RDexport	7.106	3.36 × 10^−14^	5.177	3.71 × 10^−9^	1.105	2 × 10^−16^	0.940	5.97 × 10^−14^
RDincome	8.534	2 × 10^−16^	4.772	2 × 10^−16^	4.373	2 × 10^−16^	8.666	2 × 10^−16^
lDurban	8.797	1.31 × 10^−13^	7.957	5.42 × 10^−4^	5.320	5.71 × 10^−10^	8.476	1.48 × 10^−8^
lDold	4.006	2 × 10^−16^	2.920	1.39 × 10^−3^	2.604	9.54 × 10^−6^	4.556	7.46 × 10^−13^

From the non-linear results of the national model fitting in Figure 1, the proportion of government health expenditure has a non-linear influence on economic development. With the gradual increase in government health expenditure, the extent of economic development also gradually increases. The proportion of fixed assets investment also has a non-linear effect, in the form of a convex curve; however, it shows a negative trend with respect to economic development, and the curve is relatively flat. The trend of the proportion of exports declines, then rises and then declines again; the overall performance shows a negative trend, and the decline is flat. The proportion of residents' income shows a “*V*-shaped” impact on economic development, with the impact on economic development first declining and then rising. The level of urbanization presents a wave-shaped relationship with economic development. The impact of the rising shape has a positive effect on economic development. The impact of the proportion of the elderly population on economic development shows a relatively flat inverted “*U*-shaped” relationship, having a positive impact after a negative impact on economic development.

From the results of the non-linear effects on the eastern, central and western regions in Figures 4–6, in the eastern region, the impact of the proportion of government health expenditure shows a wavy upward trend with respect to economic development. The impact of the proportion of fixed assets investment on economic development shows a wavy decline. The trend of the export ratio is basically the same as that for the nation. It has the trend of first decreasing, then rising and then decreasing again, and the overall trend is downward. The impact of the proportion of residents' income on economic development shows a convex downward curve. The impact of the level of urbanization on economic development shows a wavy downward trend. The proportion of the elderly population is basically consistent with the trend of the non-linear influence for the nation, presenting a flat inverted “U-shaped” relationship with economic development.

In the central region, the proportion of government health expenditure shows a positive trend with respect to economic development, and the upward trend is faster. The proportion of fixed assets investment shows a rapid wave-shaped downward trend with respect to economic development, showing a nearly linear decline curve with regard to economic development. The impact of the proportion of residents' income on economic development first declines slowly and then declines rapidly. The impact of the level of urbanization on economic development shows an “*M*-shaped,” first rising, then falling, then rising after the decline. The proportion of the elderly population showed a rapid rise after the rapid rise in the central region, and the positive impact on economic development was first slowed down. In the western region, the impact of the proportion of government health expenditure on economic development shows a convex upward curve, showing a slow promotion effect on economic development. The impact of the proportion of fixed assets investment on economic development shows a relatively flat “*U*-shaped” trend. The proportion shows a nearly linear downward trend. The impact of the proportion of residents' income on overall economic development shows a “*V*-shaped” pattern. The urbanization level shows a wave-shaped rise in the early stage and a rapid decline in the later stage. The impact of the proportion of the elderly population on economic development is consistent with the eastern region, presenting a flatter inverted “*U*-shaped” trend.

#### Comprehensive Results

**Figure 7:**
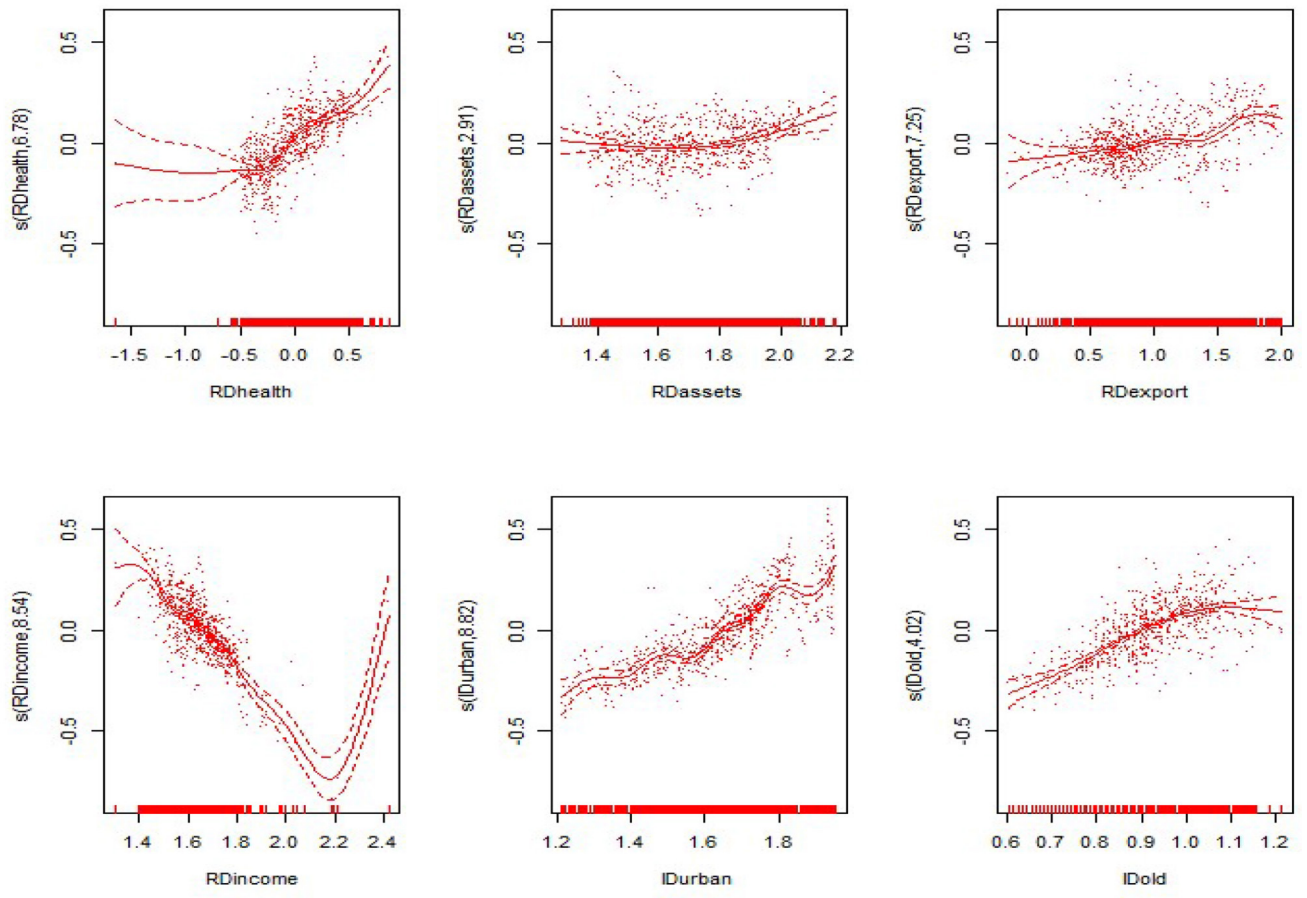
Comprehensive effects of the explanatory variables on per capita GDP in the nation.

**Figure 8:**
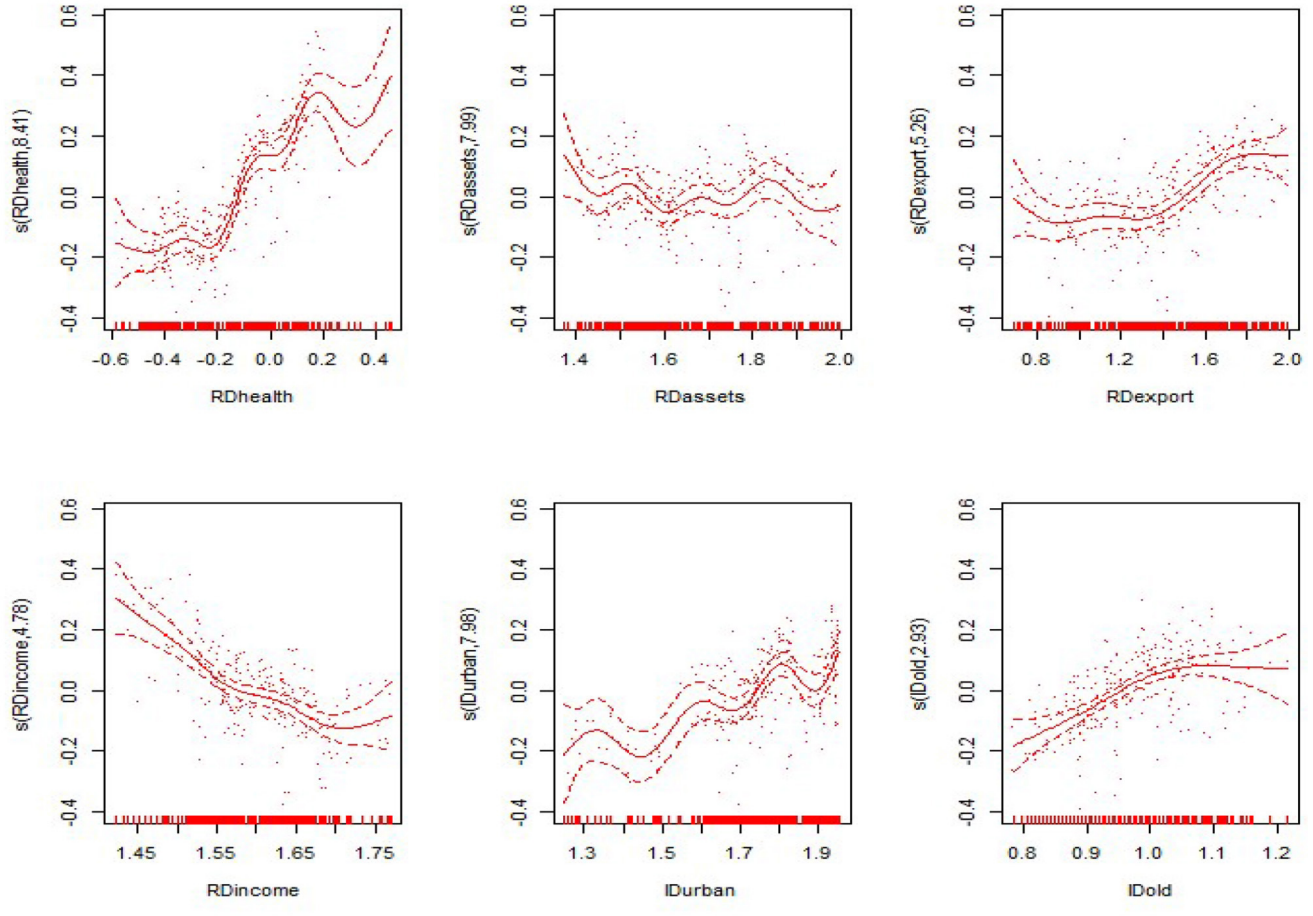
Comprehensive effects of the explanatory variables on per capita GDP in the eastern region.

**Figure 9:**
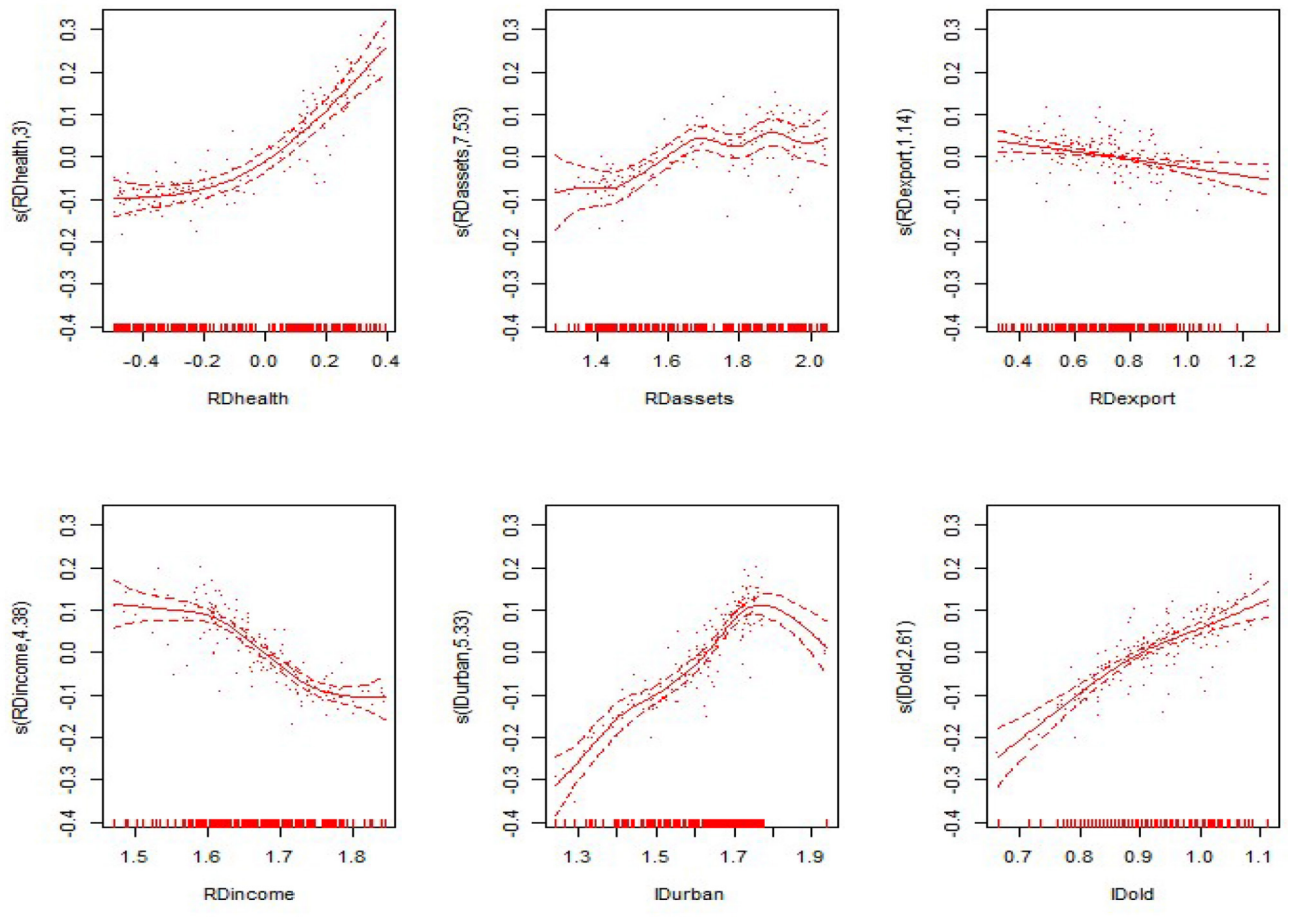
Comprehensive effects of the explanatory variables on per capita GDP in the central region.

**Figure 10:**
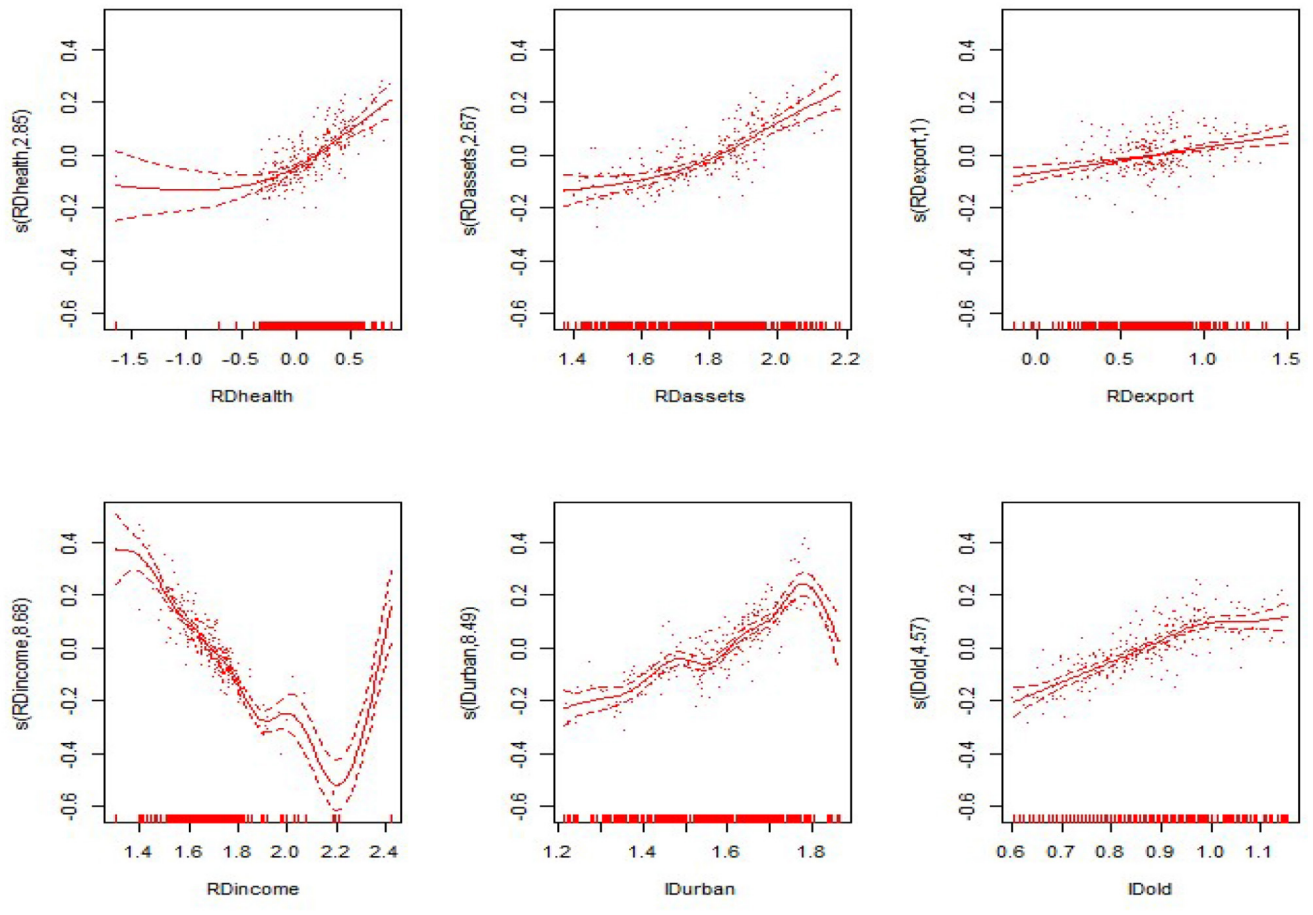
Comprehensive effects of the explanatory variables on per capita GDP in the western region.

The two subsections above show the linear and non-linear effects of each explanatory variable on economic development, but the final impact of each explanatory variable on economic development requires a comprehensive effect analysis. The following is a summary and discussion of the trends of the comprehensive effects of the explanatory variables on economic development nationwide and in the eastern, central and western regions. The trends are shown in Figures 7–10.

Regarding the national comprehensive effects of various variables, the linear effects and non-linear effects of the proportion of government health expenditure positively promote economic development. However, the non-linear impact is significant, the linear effect is not significant, and the comprehensive impact has a significant non-linear impact. With the increasing proportion of government health expenditure, the economic development level shows a convex curve growth trend, indicating that the proportion of government health expenditure has a positive impact on economic development. The proportion of fixed assets investment shows a gradual “convex” curve growth trend, indicating that with the increase in the proportion of fixed assets investment, the level of economic development slowly increases. The proportion of exports shows a wave-shaped upward trend. With the increase in the proportion of exports, the level of economic development generally shows an upward trend. The proportion of residents' income shows a positive “*V*-shaped” effect on the level of economic development, indicating that with the increase in the income level of residents, the level of economic development shows a downward trend and then an upward trend, according to the current situation of China's economic development. The reason may be that the living standard of early residents is not high. With the increase in income, early residents will use a large amount of income to save, and the consumption level will not be high, which will have a negative impact on economic development. When the income of residents reaches a certain level, it is no longer necessary for them to use more savings for their own protection. They will continuously expand their scope of consumption, increase consumer spending, and begin to play a positive role in promoting economic development. The level of urbanization shows a wavy upward trend with respect to economic development, indicating that the improved urbanization level positively promotes the level of economic development. The proportion of the elderly population shows a flat inverted “*U*-shaped” curve with respect to economic development. With the increase in the proportion of the elderly population, the level of economic development shows a trend of growth and then decline. The reason may be that the increase in the elderly population in the previous period drives the development of the elderly care industry. The effect of industrial development on the economy is higher than the negative impact of the elderly population on the economy. Therefore, the overall impact is positive. As the proportion of the elderly population increases, the economic burden continues to increase, and the impact of the elderly care industry on economic development tends to be saturated. The comprehensive effects of the two show a negative impact on economic development.

Regarding the comprehensive effects in the eastern region, the proportion of government health expenditure has a wavy upward impact on economic development. As the proportion of government health expenditure continues to increase, economic development shows a positive growth trend. The non-linear impact of the proportion of fixed assets investment is negative, while the comprehensive impact is wavy; there is no obvious upward trend. The reason may be that the eastern region's fixed assets investment level is already high; thus, increases in the proportion of fixed assets investment do not exert a significant impact. The proportion of exports increases, and the level of economic development shows a wavy upward trend, indicating that increases in the proportion of exports still have a significant positive effect on economic development in the eastern region. As the proportion of residents' income increases, the level of economic development also shows a *V*-shaped trend. In the eastern region, residents' income has been heavily invested in wealth management and investment markets. There are no savings, and there is no large amount of consumption. Therefore, the proportion of residents' income in the eastern region will not have a high promotion effect on the economy. The level of urbanization in the eastern region increases, and the level of economic development shows a clear wave-like upward trend, indicating that urbanization has a positive effect on economic development. The proportion of the elderly population is basically consistent with the national trend, and the initial stage has a positive impact on economic development. As the role of industrial promotion has gradually weakened, the burden on the economic development of the elderly population has gradually increased, and this economic development has gradually exerted a negative impact.

Regarding the central and western regions, the proportion of government health expenditure increases, and economic development shows a non-linear growth trend, indicating that the impact of government health expenditure on economic development has significant non-linear effects nationwide and in the eastern, central and western regions. The proportion of fixed assets investment in the central and western regions has a significant non-linear impact that positively drives overall economic development. The proportion of exports in the central and western regions has different effects on economic growth. It has a linear negative impact in the central region. The reason may be that the high quality and service required for exports in the central region cannot be maintained. The promotion capacity needs to be strengthened. Forcing the level of exports to increase will consume a large amount of resources such as manpower, material resources and capital. It will lead to negative economic development. The linear impact of the proportion of exports in the western region is greater than the non-linear impact. It has a linear positive impact in the western region. The reason may be that the western region itself has some naturally suitable export products and provides policy and financial support, creating better export conditions. The proportion of exports will significantly stimulate regional economic development. The impact of the proportion of residents' income is also different in the central and western regions. It has a negative impact in the central region because the economic development level in the central region is still not very high overall and has not yet reached the standard of living for free consumption. Therefore, the income of most residents enters savings and cannot contribute to economic growth. In the western region, the impact of the proportion of residents' income is basically consistent with the national trend, and the overall trend is “*V*-shaped.” The trends of urbanization in the central and western regions are consistent. With the improvement of the urbanization level, the level of economic development first rises and then declines. With the strengthening of urbanization in the early stage, urban industrial agglomeration has accelerated industrial development, improved labor and capital agglomeration, and effectively promoted rapid economic development. High urbanization will also result in a series of problems such as environmental pollution, ecological damage, and space congestion, which will have a negative impact on economic development. The proportion of the elderly population in the nation and in the eastern, central and western regions has the same impact on economic development, first growing and then falling.

Based on the above results, the proportion of government health expenditure does not have purely linear effects on economic development. Instead, it has a combination of linear and non-linear effects. It is necessary to analyze its comprehensive effects. Regardless of whether a region or the nation as a whole is being analyzed, the proportion of government health expenditure have a non-linear positive impact on economic development. Similarly, the proportion of fixed assets investment, the proportion of exports, the proportion of residents' income, the level of urbanization, and the proportion of the elderly population have a non-linear effect on economic development. However, according to the actual situation of different regions, the impact is different.

### Model Check

**Figure 11:**
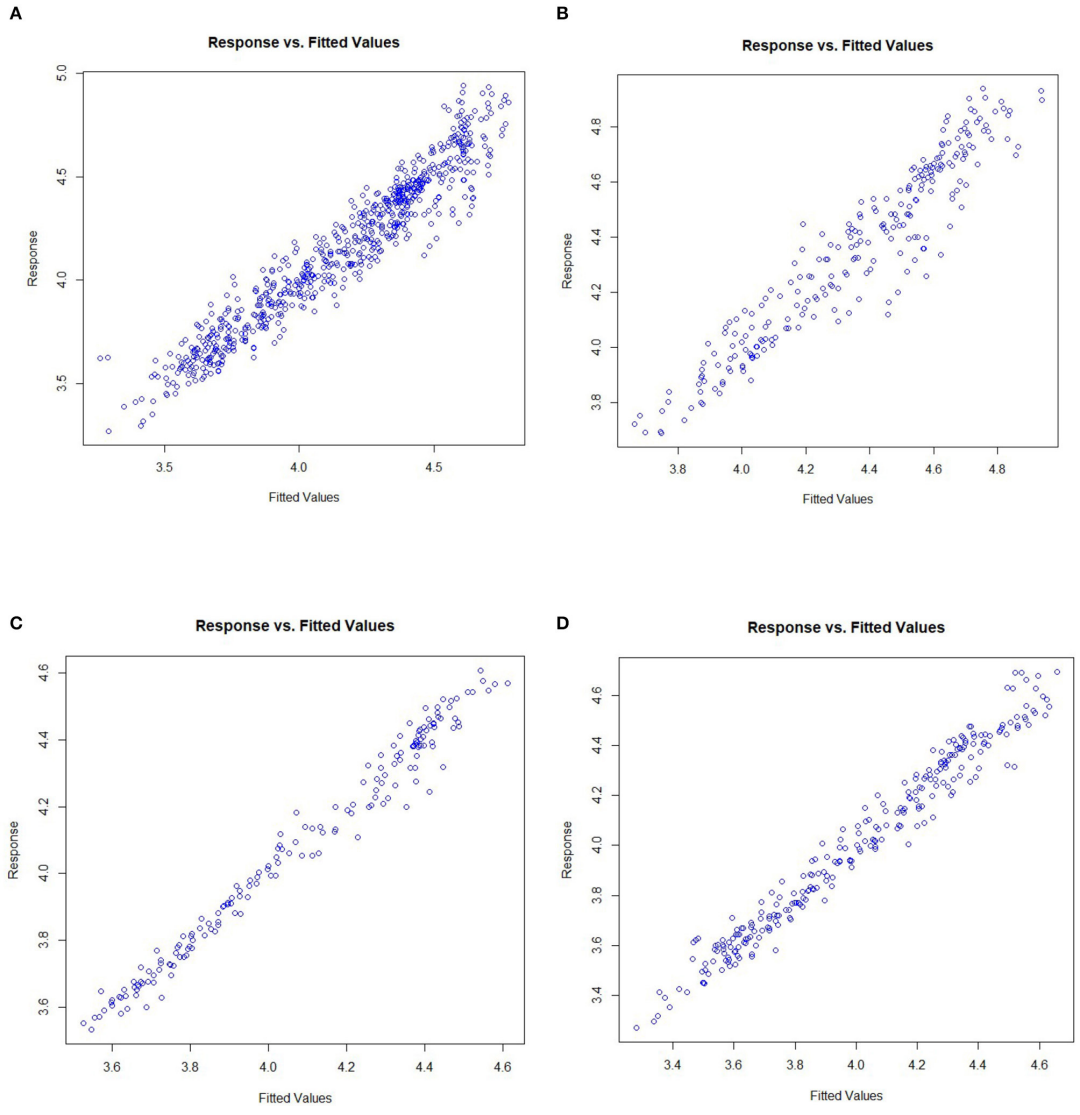
Plots of the response values vs. the fitted values for the nation and the eastern, central, and western regions. **(A)** Nation, **(B)** Eastern region, **(C)** Central region, and **(D)** Western region.

To prove the model effect, the paper tests the relationship between the fitted values and the response values. Plots of the response values vs. the fitted values for the nation and the eastern, central and western regions are shown in Figure 11.

It can be seen from Figure 11 that there is a clear linear relationship between the fitted values and the response values in the four plots, indicating that the model has a good fitting effect and that the estimation results have high credibility.

To further test the fitting effect of the model, the residual sum of squares of the non-parametric additive model is compared with the ordinary linear model, as shown in Table 6. It can be seen from Table 6 that the residual sum of squares of the non-parametric additive model is significantly smaller than that of the ordinary linear model, indicating that the model is more accurate than the estimation results of the ordinary linear model.

**Table 6:** Residual sum of squares between the non-parametric additive model and the ordinary linear model.

	**Non-parametric additive model**	**Ordinary linear model**
Nation	7.0816	11.1637
Eastern region	2.3643	4.5140
Central region	0.3247	0.7395
Western region	1.0386	2.6146

Based on the above test, it can be seen that the non-parametric additive model can analyze the linear relationship and the non-linear relationship between the variables separately. Thus, the influence mechanism between the variables is clearer and has a more superior estimation effect. Therefore, the model is used to analyze the effects of government health expenditure on economic development and is more scientific and accurate.

### Endogenous Test

To deal with the endogeneity problem, the paper tests the endogeneity by 2sls. The results are shown in Table 7.

**Table 7:** Endogenous test results.

	**Nation**		**Eastern region**		**Central region**		**Western region**	
	**Coefficients**	***P*-value**	**Coefficients**	***P*-value**	**Coefficients**	***P*-value**	**Coefficients**	***P*-value**
RDhealth	0.4627	0.000	0.9251	0.000	0.4308	0.000	0.2557	0.000
RDassets	0.0168	0.802	−0.1921	0.027	0.1990	0.054	0.5935	0.000
RDexport	0.1154	0.000	0.2320	0.000	0.1048	0.000	0.0103	0.688
RDincome	0.1363	0.000	−1.2982	0.000	0.9498	0.000	0.8995	0.000
lDurban	0.8952	0.000	0.3975	0.000	0.6118	0.000	0.7991	0.000
lDold	0.8972	0.000	0.7316	0.000	0.7823	0.000	0.6715	0.000
intercept	3.6006	0.000	5.1672	0.000	3.7294	0.000	2.5803	0.000
Underidentification test	140.749	0.000	71.004	0.000	28.965	0.000	61.809	0.000
Cragg-Donald Wald F statistic	1,134.247	640.218	251.662	146.368
Hansen J statistic	0.549	0.4588	0.014	0.9065	3.694	0.0546	2.889	0.0892

It can be seen from Table 7 that the effects of the estimated results in each region is basically the same as the previous results. Under identification test and Hansen *J*-test indicate that the over-identification test is past. And Cragg-Donald Wald *F* statistic test shows that the weak instrumental variable test is significantly passed. So the test result is valid.

## Conclusions and Recommendations

### Conclusions

This study attempts to analyze the linear, non-linear and comprehensive effects of government health expenditure on the economic development of the nation and the eastern, central and western regions of the nation by using a non-parametric additive model. The results show the following: (a) based on the different geographic entities analyzed, i.e., the nation and the eastern, central and western regions, the linear impact of the proportion of government health expenditure on economic development is positive nationwide and in the western region, negative in the eastern and central regions, and significant only in the western region. The proportion of government health expenditure has a significant non-linear impact on economic development nationwide and in the eastern, central and western regions. The comprehensive effect of combining its linear and non-linear effects shows significant non-linear positive effect nationwide and in all regions. (b) The impacts of the proportion of fixed assets investment, the proportion of exports, the proportion of residents' income, the level of urbanization, and the proportion of the elderly population on economic development are significantly positive nationwide and in the eastern, central and western regions. In addition, their non-linear influences in different regions vary depending on regional differences. Regarding the comprehensive impact of different variables on economic development, the proportion of fixed assets investment has a positive non-linear effect nationwide and in all regions; the proportion of exports has a positive non-linear effect nationwide and in the eastern region. However, in the central and western regions, it has a nearly linear negative impact. The proportion of residents' income has a positive “*V*-shaped” effect nationwide and in the western region, and it has a non-linear negative impact in the eastern and central regions. The urbanization level shows a non-linear positive impact nationwide and in the eastern region. The trend in the central and western regions shows first growth and then decline. The proportion of the elderly population in the nation and in the eastern, central and western regions shows a first rapidly positive and then a flat positive impact on economic development. (c) The non-parametric additive model does not perform a simple analysis of the influence between variables. However, it can analyze the linear influence and the non-linear influence separately, which can help us more clearly understand the conduction path between variables. Compared with ordinary linear regression, it is more scientific and accurate.

### Recommendations

Currently, the challenge of economic growth is being faced both internationally and domestically. Economic growth has slowed down. Some countries have even begun to experience negative growth. China's economy has also begun to undergo structural transformation from the initial “extensive” rapid growth, and it is necessary to maintain steady economic development. It is necessary to adjust the various factors that promote the stable development of the economy in a scientific and rational way. Based on the conclusions of this research, the following policy implications can be obtained:

First, whether for the whole country or for different regions, the government should promote the further development of the economy by increasing the proportion of government health expenditure to GDP. By increasing government health investment, the government can increase investment in medical and health resources and improve the quality of medical and health personnel. To improve the service level of medical institutions and medical personnel, the government should promote the improvement of the health of human capital and provide high-quality human resource reserves for China's economic development.

Second, we must promote social and business investment in fixed assets, promote product exports, promote innovation and entrepreneurship to create more jobs for the society, continuously increase residents' income, promote urbanization, and create a good development environment for the rise of the elderly care industry and related industries, which should then be promoted. In addition, we must create jobs for the elderly population and reduce the personal and national economic burden of the elderly population after retirement.

Third, the economic development of the eastern, central and western regions can be increased by increasing investment in fixed assets. In addition, for the eastern region, relevant policies can be introduced to stimulate exports and expand urbanization by government. For the central region, exports should not be pursued excessively. The level of urbanization should be expanded, and the income level of residents should be appropriately raised to develop the economy. However, in the process of promoting urbanization, the complex impact of urbanization must also be taken into account. For the western region, government should continue to adhere to the western support plan, and the problem of slow economic development should be solved by promoting exports and increasing residents' income.

## Data Availability Statement

The original contributions presented in the study are included in the article/supplementary material, further inquiries can be directed to the corresponding author.

## Author Contributions

YW and CT contributed to conception and design of the study. QX organized the database. YW performed the statistical analysis and wrote the first draft of the manuscript. CT and QX wrote sections of the manuscript. All authors contributed to manuscript revision, read, and approved the submitted version.

## Funding

This research was funded by the Bidding Project of National Social Science Foundation of China, Research on the Regional Economic Growth Effect of the Agglomeration of China's Pharmaceutical Manufacturing Industry from the Perspective of Resource Mismatch (Grant No. 20ATJ003).

## Conflict of Interest

The authors declare that the research was conducted in the absence of any commercial or financial relationships that could be construed as a potential conflict of interest.

## Publisher's Note

All claims expressed in this article are solely those of the authors and do not necessarily represent those of their affiliated organizations, or those of the publisher, the editors and the reviewers. Any product that may be evaluated in this article, or claim that may be made by its manufacturer, is not guaranteed or endorsed by the publisher.
